# IGL-CCND1重排阳性套细胞淋巴瘤1例报告并文献复习

**DOI:** 10.3760/cma.j.issn.0253-2727.2023.07.015

**Published:** 2023-07

**Authors:** 迎春 郑, 佳炜 赵, 旭 郭, 树华 易, 媛 陶, 承文 李

**Affiliations:** 中国医学科学院血液病医院（中国医学科学院血液学研究所），实验血液学国家重点实验室，国家血液系统疾病临床医学研究中心，细胞生态海河实验室，天津 300020 State Key Laboratory of Experimental Hematology, National Clinical Research Center for Blood Diseases, Haihe Laboratory of Cell Ecosystem, Institute of Hematology & Blood Diseases Hospital, Chinese Academy of Medical Sciences & Peking Union Medical College, Tianjin 300020, China

套细胞淋巴瘤（MCL）是一种具有独特生物学特征的B细胞非霍奇金淋巴瘤（NHL），大约95％的MCL患者存在特征性的染色体易位t（11;14）（q13;q32），由此产生免疫球蛋白重链-细胞周期蛋白1（IGH-CCND1）基因重排，从而导致了cyclin D1异常过度表达 [1]。但涉及免疫球蛋白轻链和其他伙伴基因的易位较为罕见，我们报道1例IGL-CCND1重排阳性的MCL患者，并进行相关文献复习，以更好地了解其临床和实验室特征。

## 病例资料

患者，女，51岁，以“ 乏力2个月，右下颌疼痛10 d”为主诉入院。查体：中度贫血貌，周身皮肤无皮疹、黄染、出血点，浅表淋巴结无肿大。咽部无充血，扁桃体无肿大。胸骨无压痛，腹部平坦，无压痛及反跳痛，肝肋缘下未触及，脾肋缘下8 cm可及，质硬。双下肢无水肿。腹部超声：肝多发囊肿，脾重度肿大，胆胰未见异常。CT平扫：颈、胸、腹、盆腔多发淋巴结，部分肿大，脾大。血常规：WBC 2.68×10^9^/L，RBC 1.79×10^12^/L，HGB 61 g/L，PLT 110×10^9^/L。血浆生化：白蛋白 20.1 g/L，LDH 87.5 U/L，β_2_微球蛋白1.99 mg/L。骨髓细胞形态学：增生活跃，粒系占6.5％，红系占1％，粒∶红为6.5∶1，淋巴细胞比例增高，易见不典型淋巴细胞，全片共见巨核细胞24个；外周血淋巴细胞比例增高，易见不典型淋巴细胞。骨髓活检：HE及PAS染色示送检骨髓增生极度活跃（约90％），异常淋巴细胞增生（40％～50％），散在或灶性分布，胞体小至中等大，胞质量少，胞核椭圆形或略不规则，核染色质粗；粒红系各阶段细胞可见，均以中幼及以下阶段细胞为主，巨核细胞不少，分叶核为主；网状纤维染色（MF-1级）。免疫组化显示肿瘤细胞：CD20（+），PAX5（+），CD5（少量弱+），CD23（少量弱+），cyclin D1（部分+），SOX11（−），LEF1（−），CD3（−），CD10（−）（图1）。行颈深部淋巴结切除术，术中取出1枚肿大淋巴结送病理及流式细胞术免疫分型检查。颈部淋巴结切检：送检淋巴结结构破坏，异常淋巴细胞弥漫增生，胞体小至中等大，胞质量少，胞核略不规则，核染色质粗。免疫组化显示肿瘤细胞：CD20（+），PAX5（+），CD5（部分弱+），cyclin D1（+），CD23（部分+），BCL2（+），Ki-67阳性率20％～30％，CD3（−），CD10（−），SOX11（−），LEF1（−），MUM1（−），CD25（−），CD103（−）；CD21（FDC+）（图2）。原位杂交：EBER阴性。淋巴结流式细胞术免疫分型：异常细胞群约占有核细胞的77.03％，强表达FMC7，表达CD19、CD20、CD200、CD22、Lambda，弱表达CD5、CD79b、CD23、CD11c、CD81，不表达CD10、CD25、CD103、CD38、sIgD、sIgM、Kappa。骨髓及外周血免疫分型与淋巴结免疫表型一致。骨髓染色体核型分析双体系培养共分析25个分裂象，初步结果为46,XX,del（11）（q13q22）,del（22）（q11.2）[5]/46,XX[20]。其中克隆性异常11q−、22q−均见于72 h磷酸胞苷酰寡脱氧核苷酸（CPG-ODN）刺激法培养结果，24 h短期培养法均为正常核型。骨髓间期细胞荧光原位杂交检测IGH-CCND1基因重排阴性，可见41％的细胞CCND1基因信号增加1个拷贝，信号特征为3R2G；IGH基因重排检测为阴性；ATM基因缺失阳性，信号特征为1R2G。RB-1基因、12号染色体相关CEP12基因、IGH-BCL2基因、TP53基因、IGH-CCND3基因及CCND2基因检测均为阴性。结合IGH-CCND1的FISH结果补充了CCND1基因分离探针，结果显示50％的细胞信号特征为2F1G异常信号（图3A）；同时补充IGL和IGK基因重排FISH检测，结果显示有45％细胞IGL基因重排阳性，信号特征为1F1R1G（图3B），IGK基因重排为阴性。随后通过IGL/CCND1双色双融合探针验证，结果52％细胞IGL-CCND1融合基因阳性，其中4％细胞信号特征为2F1R1G，34％细胞信号特征为1F2R2G（图3C），14％细胞信号特征为1F1R2G。其中1F2R2G提示可能存在复杂易位，又通过IGL-CCND1双色双融合探针的中期FISH进行验证，可见阳性信号特征为1F1R2G，融合信号在der（11）上（图4），结合中期FISH结果修正染色体核型异常为46，XX，der（11）t（11;22）（q13;q11.2），del（22）（q11.2）。分子遗传学方面，IGH体细胞高突变分析，IGH克隆性重排为突变型，重排类型为IGHV4-39，IGH体细胞突变率为3.7％。二代测序：拷贝数变异分析，检测到BIRC3和ATM基因拷贝数减少，BIRC3基因检测到p.Q484*突变和p.L514Afs*3突变，CXCR4基因检测到p.R334*突变。结合临床及实验室检查，本病例最终诊断为MCL。予以R-DHAP方案（利妥昔单抗、地塞米松、阿糖胞苷、顺铂）化疗，患者目前病情平稳，仍在随访中。

**图1:**
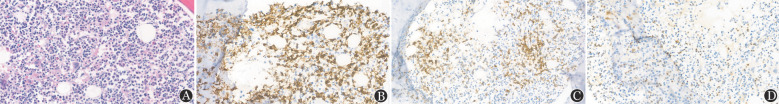
患者骨髓活检HE及免疫组化染色结果 A 骨髓活检中可见异型淋巴细胞增生，散在或簇状分布，胞体小至中等大，胞质量少，核略不规则，染色质粗（HE染色，×400）； B 免疫组化染色示肿瘤细胞CD20阳性（Elivision法，×400）； C CD5少量弱阳性（Elivision法染色，×400）； D cyclin D1部分阳性（Elivision法染色，×400）

**图2:**
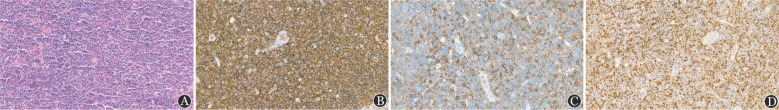
患者淋巴结切除活检HE及免疫组化染色结果 A 淋巴结结构完全破坏，异型淋巴细胞弥漫增生，胞体小至中等大，胞质量少，核略不规则，染色质粗（HE染色，×400）； B 免疫组化染色示肿瘤细胞CD20阳性（Elivision法染色，×400）； C CD5弱阳性（Elivision法染色，×400）； D cyclin D1弥漫阳性（Elivision法染色，×400）

**图3:**
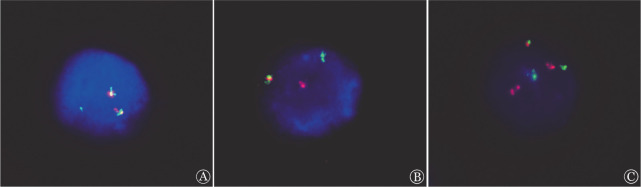
患者骨髓间期FISH结果 A CCND1（5′G /3′R）重排阳性，2F1G提示断裂点在5′端； B IGL（5′R/3′G）重排阳性，信号为1F1R1G； C IGL(G)/CCND1(R)重排阳性，信号1F2R2G提示复杂易位

**图4:**
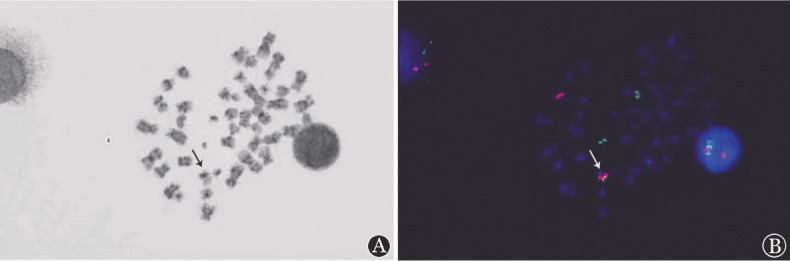
患者骨髓中期FISH结果 A 中期染色体核型； B 中期IGL(G)/CCND1(R)重排阳性，信号为1F1R2G，箭头显示融合信号在der(11)上

## 讨论及文献复习

导致癌基因激活的染色体易位是B细胞淋巴瘤的特征。这些疾病的特异性克隆性染色体异常在肿瘤发展中起着关键作用。大约95％的MCL患者存在t（11;14）（q13;q32），导致CCND1基因与IGH基因重排，促进cyclin D1蛋白的合成和过度表达。其他形态相近的淋巴瘤均不具有此特点，故通过各种方法检测t（11;14）和cyclin D1表达是MCL的诊断及与其他淋巴瘤鉴别诊断的重要手段。在染色体核型分析中，由于成熟B淋巴细胞增殖活性较低，若不加入刺激剂培养，仅有50％～65％的MCL患者可检测出t（11;14），但如果使用FISH技术对骨髓或石蜡组织标本进行检测，则可提高IGH-CCND1基因重排的检出率[2]–[3]。在本病例的遗传学分析中，染色体核型分析并未检测到t（11;14），而通过FISH我们首先检测到IGH-CCND1基因重排阴性，但可见CCND1基因信号增加1个拷贝，而IGH基因信号没有增加，结合CEP11/ATM探针检测结果，未见到额外增加的着丝粒信号，推测CCND1基因增加的额外信号并非是由11号染色体三体形成，而是可能发生了基因的扩增或重排。补充CCND1基因分离探针检测，结果显示CCND1基因重排阳性，从而证实了我们的推测；但CCND1基因重排的伙伴基因并非常见的IGH，进一步推测可能为免疫球蛋白轻链基因如2号染色体p12上的IGK或22号染色体q11上的IGL基因，进而我们又通过FISH分别检测了IGL和IGK基因重排，结果显示IGL基因重排阳性，IGK基因重排为阴性。综合上述检测结果，IGH- CCND1基因重排阴性，且CCND1基因额外增加了一个信号，IGL基因和CCND1基因重排均为阳性，提示CCND1基因可能与IGL基因发生了重排，即11号染色体和22号染色体发生了易位，与染色体核型结果中der（11）成因推测相印证。为了进一步证实这一推测，我们采用IGL /CCND1双色双融合探针进行了验证，结果显示IGL-CCND1基因重排阳性，此例患者为涉及IGL的CCND1重排阳性MCL。该例患者细胞遗传学检验思路如图5。

**图5:**
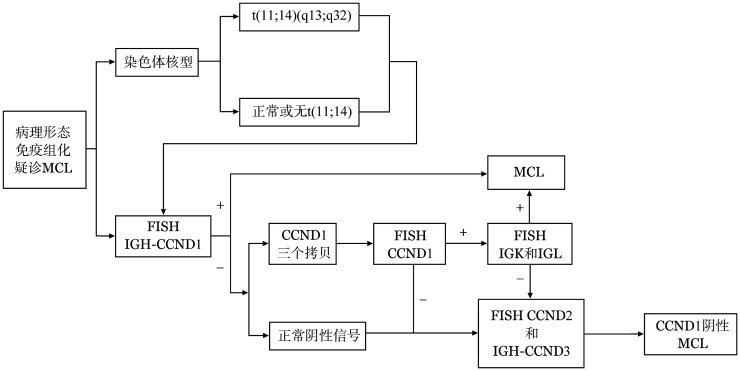
本例套细胞淋巴瘤（MCL）患者细胞遗传学检验思路流程图

据报道，在5％～10％的B细胞淋巴瘤患者中存在累及免疫球蛋白轻链位点的变异易位[4]，包括IGK（2p11.2）和IGL（22q11.2）。变异易位主要见于伯基特淋巴瘤和滤泡性淋巴瘤。一般来说，绝大多数MCL患者表现为t（11；14）（q13；q32），导致IGH-CCND1基因重排，而CCND1和免疫球蛋白kappa链（IGK）或lambda链位点（IGL）之间的易位很少被描述。据报道，涉及CCND1和IGK的非典型易位也导致cyclin D1的过度表达[5]。在不同的肿瘤中亦发现了IGL易位，在慢性淋巴细胞白血病中t（18;22）导致BCL2和IGL重排[6]，在Burkitt淋巴瘤中导致MYC和IGL重排[7]。与其他淋巴瘤相比，仅在少数MCL病例中观察到累及CCND1的变异易位。Komatsu等[8]报道了1例MCL患者，显示非典型易位t（11；22）（q13；q11），他们首先推测这种易位可能导致CCND1重排到IGL基因位点，并在随后的研究中在分子水平上证实了他们的假设[9]。WHO的分类参考了以上研究，指出了这一罕见易位的可能性。此外，Wlodarska等[5]报告了3例累及IGK位点的t（2；11）（p11；q13）变异易位MCL患者，临床表现均类似CLL，呈惰性。与t（11；14）（q13；q32）相反，t（11；22）（q13；q11）/IGL-CCND1的断裂发生在CCND1外显子5的3′非翻译区（UTR）内，其次是相同转录方向的IGL恒定基因（IGLC）[9]。另一方面，影响CCND1 基因3′ UTR的点突变、缺失或易位导致截短信使RNA（mRNA）的表达，该表达比正常转录物更稳定，从而增加CCND1的水平[10]–[13]。

迄今为止已报道4例累及IGL-CCND1的MCL患者[8],[14]–[16]，均为中老年人，女3例，男1例。免疫表型均为CD5^+^CD10^−^小B细胞淋巴瘤，1例克隆限制性表达Kappa，2例克隆限制性表达Lambda，1例Kappa、Lambda均阴性。染色体核型分析均无典型的t（11;14），而是累及11号和22号染色体，此外有3例患者伴随附加染色体异常，其中1例还可见双微体。经FISH或分子学检测证实为IGL-CCND1基因重排阳性，免疫组化示cyclin D1蛋白过表达，1例SOX11阳性，1例IGHV突变阳性。3例有淋巴结受累，1例为白血病样非淋巴结性MCL。而本例患者核型分析和FISH检测中亦缺乏典型的t（11;14），免疫组化结果显示cyclin D1阳性，与上述病例不同的是，之前报道的4例患者中有3例是经典型的MCL，1例是白血病样非淋巴结性MCL（惰性MCL），而本例是经典型MCL，有淋巴结的受累，又有骨髓受累及白血病样表现。

综上所述，本研究为中国患者中IGL-CCND1阳性MCL的首次报告。累及IGL-CCND1的MCL患者较为罕见，既可以有经典型MCL，又有白血病样非淋巴结性MCL，既有典型的MCL特征，如CCND1易位、附加染色体异常（3号染色体三体、1p21的断裂及17p的缺失）和cyclin D1过度表达，但也发现了一些非典型特征，如双微体的存在和IGHV突变。这些病例均缺乏t（11;14），但存在cyclin D1的过表达，临床、组织学、流式细胞学和免疫组化结果与MCL的诊断相符。涉及免疫球蛋白轻链易位的MCL的资料很少，也没有标准治疗方案。但大多相对惰性，临床病程缓慢，化疗有效，存活时间长。结合已报道的4例病例及本病例，推测累及免疫球蛋白轻链的MCL可能会经历一个类似于经典的具有t（11；14）的MCL白血病非淋巴结转移的过程。临床医师应意识到MCL中存在变异易位，以免混淆和误诊。本病例显示了细胞遗传学分析在MCL诊断及鉴别诊断中的重要性。随着有效刺激剂的使用，能够获得足够的中期细胞克隆，提高了染色体异常的检出率。使用融合探针及分离探针的间期和中期细胞FISH检测相结合是MCL诊断的最佳方法，以避免漏检。
